# Minimally invasive thoracic surgery: beyond the access route – have we truly become minimally invasive?

**DOI:** 10.31744/einstein_journal/2026Suppl_3ED0001

**Published:** 2026-06-19

**Authors:** Ricardo Mingarini Terra

**Affiliations:** 1 Hospital Israelita Albert Einstein São Paulo SP Brazil Hospital Israelita Albert Einstein, São Paulo, SP, Brazil.

Over the past three decades, thoracic surgery has undergone one of the most remarkable transformations in its history. The transition from thoracotomy to video-assisted thoracic surgery (VATS), followed by the widespread adoption of robotic-assisted thoracic surgery (RATS), has dramatically reduced surgical trauma while maintaining oncological efficacy and procedural safety.^(1,2)^ Smaller incisions, enhanced visualization, superior dexterity, and improved precision have translated into less postoperative pain, faster recovery, and shorter hospital stays. Few innovations have impacted our specialty as profoundly as minimally invasive surgery.

The adoption of robotic surgery has accelerated this evolution even further. Robotic platforms have expanded the indications for minimally invasive approaches, allowing surgeons to perform increasingly complex procedures such as anatomical segmentectomies, bronchovascular reconstructions, and advanced mediastinal resections through small incisions.^(2)^ As demonstrated by our own experience with one thousand consecutive robotic thoracic procedures, increasing expertise is associated with substantial reductions in complications and conversions, even as more complex and higher-risk patients are offered surgical treatment. Technical barriers that once limited minimally invasive surgery are progressively disappearing.

## Yet an important question remains. Can a procedure truly be considered minimally invasive if only the incision is minimal?

A robotic lobectomy performed through four 8-mm ports cannot be considered fully minimally invasive if it is followed by routine intensive care admission, prolonged chest tube drainage, delayed mobilization, multiple unnecessary investigations, and several days of hospitalization. While we have successfully minimized the surgical access, we have not always minimized the overall burden of treatment imposed on the patient.

Historically, postoperative pathways were designed for patients undergoing thoracotomy. These practices were justified by the significant physiological insult associated with open surgery. However, modern minimally invasive techniques have fundamentally changed the postoperative trajectory of many patients. Continuing to apply traditional perioperative practices to contemporary surgical procedures may prevent patients from fully benefiting from the advances achieved in the operating room.

This realization has given rise to one of the most important developments in modern perioperative care: Enhanced Recovery After Surgery (ERAS). The ERAS Society and the European Society of Thoracic Surgeons have established evidence-based recommendations that encompass the entire perioperative journey, including patient education, prehabilitation, nutritional optimization, multimodal analgesia, minimization of invasive devices, early mobilization, and standardized postoperative care pathways.^(1)^

Importantly, ERAS is not simply a protocol. It is a philosophy of care. The goal is not merely earlier discharge, but safer, faster, and more predictable recovery. Early discharge should be viewed as the consequence of successful recovery rather than the primary endpoint.

At *Hospital Israelita Albert Einstein*, we recently implemented a multidisciplinary accelerated recovery program for thoracic surgical patients based on ERAS principles. The program integrates preoperative education, respiratory prehabilitation, standardized anesthesia protocols, specialized nursing support, telemonitoring, and structured postoperative follow-up. The results have been encouraging, demonstrating meaningful reductions in hospital length of stay without increases in complications, reoperations, or readmissions. Equally important, patient satisfaction remained exceptionally high. These findings reinforce the concept that perioperative pathways can be redesigned safely when supported by multidisciplinary collaboration and evidence-based protocols.

However, one of the most important lessons from our experience is that the barriers to recovery are no longer primarily technical. Increasingly, they are cultural.

The management of chest tubes provides an illustrative example. Robust evidence demonstrates that chest drains can often be removed safely with substantially higher drainage volumes than traditionally accepted, provided that air leaks are absent and patients remain clinically stable.^(3)^ Similarly, routine chest radiographs after chest tube removal rarely alter management in asymptomatic patients. Despite this evidence, many patients continue to experience delayed discharge after fulfilling all objective criteria for hospital release.

Recent analyses from our institution have shown that postoperative complications and procedural complexity remain important determinants of prolonged hospitalization, as expected. However, among patients who achieve predefined recovery milestones, variations in discharge timing are frequently influenced by surgeon preference, institutional habits, and risk perception rather than objective clinical factors. This observation highlights a critical reality: the limiting factor is often no longer physiology, but culture.

This phenomenon is not unique to thoracic surgery. Throughout medicine, technological innovation frequently outpaces behavioral change. New surgical platforms, digital drainage systems, advanced stapling devices, and artificial intelligence are rapidly incorporated into clinical practice. Yet practices rooted in previous eras often persist despite accumulating evidence supporting alternative approaches.

As robotic surgery continues to evolve, discussions frequently focus on newer platforms, enhanced imaging systems, artificial intelligence, and increasing technical sophistication. These advances build upon the growing body of evidence supporting robotic approaches, including randomized data demonstrating the safety and effectiveness of robotic-assisted lobectomy compared with video-assisted techniques.^(4)^ Such developments are exciting and will undoubtedly contribute to future progress. Nevertheless, innovation should not be confined to the operating room. A patient who returns home safely on the first or second postoperative day after a robotic anatomical lung resection may derive a greater tangible benefit than that provided by any isolated technological advancement.^(5)^

The future of minimally invasive thoracic surgery will therefore be defined not only by how we operate, but also by how we prepare patients for surgery, manage their recovery, and facilitate their return to normal life. Technical excellence, complication avoidance, evidence-based perioperative care, and cultural transformation must work together to achieve this objective.

As we present the abstracts from the 5th International Congress on Robotic Thoracic Surgery in this supplement, it is clear that our specialty continues to innovate at an extraordinary pace. Yet perhaps the next frontier is not another technological breakthrough. Perhaps it is ensuring that every patient fully benefits from the innovations we already possess. Minimally invasive thoracic surgery must move beyond the access route. It must encompass the entire patient experience.

## Data Availability

The content is already available.
